# Operation time and outcomes in distal radius fracture fixation: a retrospective analysis of single- vs. dual-surgeon procedures in 163 cases

**DOI:** 10.1186/s12891-025-09359-4

**Published:** 2025-11-25

**Authors:** Daniel Muder, Marcus Sagerfors, Daniel Reiser

**Affiliations:** 1 Department of Surgical Sciences/Orthopaedics & Hand Surgery, Uppsala University, Entrance 70, Uppsala, SE-75185 Sweden; 2 Center for Clinical Research Dalarna, Uppsala University, Nissers väg 3, Falun, SE-79182 Sweden; 3 Department of Orthopedics and Hand Surgery, Faculty of Medicine and Health, Örebro University, Örebro, SE-70182 Sweden

**Keywords:** Distal radius fracture, Operative treatment, Surgical efficiency, Operation time

## Abstract

**Background:**

Distal radius fractures (DRFs) are among the most common orthopedic injuries requiring surgical intervention. Understanding whether single- or dual-surgeon setting correlates to operation time, radiological results, or complication rates is essential for optimizing resource allocation and training strategies in clinical settings.

**Methods:**

All DRFs treated operatively with volar plate fixation in 2023 were included in the study. Data were collected on the number of surgeons involved in each procedure and their respective levels of experience. These variables were analyzed in relation to operative time, radiological outcomes, postoperative complications, and AO classification.

**Results:**

The study cohort included 163 operatively-treated DRFs with a mean follow-up of 15 months (range: 9–20). The most common fracture types were AO types A (*n* = 80, 49%) and C (*n* = 80, 49%). Of all procedures, 45% (*n* = 73) were performed by a single surgeon, while 55% (*n* = 90) involved two surgeons. Operative time was significantly shorter when surgeries were performed by a single surgeon. Radiological outcomes and complication rates did not differ significantly based on the number of surgeons involved, irrespective of their experience level. When less experienced surgeons were excluded and only more complex fractures were analyzed, the trend toward shorter operative time with a single surgeon persisted, though the difference did not reach statistical significance.

**Conclusions:**

The involvement of two surgeons does not necessarily correlate to operative time in the treatment of DRFs. Furthermore, radiological outcomes and complication rates appear unaffected by the number of surgeons. These findings may support more efficient allocation of surgical resources in hospital settings. The impact and importance of teaching and training in this context warrants further investigation.

## Background

Distal radius fractures (DRFs) are among the most frequently encountered injuries in orthopedic trauma, and account for a substantial proportion of surgical interventions, particularly in adults and the elderly [1–3]. Operative treatment is commonly indicated in cases of displaced or unstable fractures, with volar plate fixation being the most widely used technique [4]. While numerous studies have investigated factors influencing clinical and radiological outcomes following DRF surgery—such as patient age, fracture classification, surgical technique, and timing—there remains limited evidence regarding the role of surgical team composition, particularly the number and experience level of operating surgeons [5–7].

In teaching hospitals and academic centers, procedures are often performed by teams consisting of both experienced consultants and residents in training. This practice supports the dual goals of patient care and surgical education. However, the impact of involving multiple surgeons in a single procedure on operative efficiency and patient outcomes remains poorly defined. It is commonly assumed that the presence of an additional surgeon, such as a senior hand surgeon assisting or supervising a resident, may enhance surgical workflow, reduce operative time, and improve outcomes through shared decision-making and technical support [8]. Conversely, it is also plausible that increased personnel could contribute to longer operative times due to the coordination required between team members, especially in training environments [9].

Assessing whether the number of surgeons involved in DRF surgery has any measurable effect on key parameters such as operative time, radiographic alignment, or complication rates is essential for optimizing surgical planning and resource allocation. In an era of increasing pressure on healthcare systems to efficiently deliver high-quality care, such insights are valuable in guiding staffing strategies in the operating theatre [10, 11]. An improved understanding of these dynamics may also contribute to the ongoing discussion about balancing effective surgical training with patient safety and operational efficiency. Studies suggest that shorter operation times are associated with fewer complications and reoperations, highlighting the importance of further exploring the factors that may influence surgical duration [12].

This study aimed to evaluate the relationship between the number of surgeons involved in DRF procedures and operative time, radiological outcomes, and postoperative complications, while also considering the surgeons’ levels of experience and the fracture type. The findings may help inform evidence-based decisions regarding surgical team composition in the operative management of DRFs.

## Methods

### Study design and setting

The study was approved by the National Ethical Review Board (8 October 2024; ref: 2024–03967-01) and conducted according to the Declaration of Helsinki. Given the study design and the approval from the National Ethical Review Board, EPM – Etikprövningsmyndigheten (October 8th 2024; ref: 2024–03967-01) individual patient consent was not required. Instead, each patient was individually contacted by letter and asked to return an opt-out form within four weeks. All patients who returned the opt-out form were excluded from the study. A retrospective chart review was performed at a Swedish regional hospital: a secondary care institution serving a population of approximately 230 000 people. Patients were identified using the hospital’s electronic medical records system by querying the International Classification of Diseases, 10th Revision (ICD-10) codes S52.5 (fracture of the distal radius) and NCJ69 (osteosynthesis with plates and screws for fixation). The search included all patients who underwent surgical treatment for distal radius fractures between 1 January 2023, and 31 December 2023. Follow-up ended on the day of revision, reported complication, death, or 25 October 2024, whichever came first. This study was registered with the National Ethical Board on October 8, 2024; Trial ID: 2024–03967-01.

### Inclusion and exclusion criteria

Inclusion and exclusion criteria are shown in Table 1.

**Table 1:** Inclusion and exclusion criteria

Inclusion criteria	Exclusion criteria
ICD-10 code S52.5 or S52.51 Operated DRF	Not DRF Not operated
Operated in 2023	Not operated in 2023
Above 18 years of age	Under 18 years of age
Radiographs available	No radiographs available

DRF Distal radius fracture, *ICD-10* International Classification of Diseases, 10th Revision

### Data collection

Demographic and clinical data were extracted from medical records, including patient age, sex, date of injury, fracture classification according to the Arbeitsgemeinschaft für Osteosynthesefragen/Orthopaedic Trauma Association (AO/OTA) system, operative reports, and details regarding the surgical team. Specifically, operation time, the number of surgeons involved, and the surgeons’ experience level as defined by Tang and Giddins [13] were recorded for each case: level 1 – non-specialist (in training or general practitioner), level 2 – specialist with limited experience, level 3 – experienced specialist (typically >5 years in practice), level 4 – highly experienced specialist, and level 5 – expert (specialist with recognized academic or technical contribution to the field). Trauma was classified as high-energy if it involved vehicles such as all-terrain vehicles, motorcycles, or snowmobiles, or resulted from a fall from a height greater than 1 m. Low-energy trauma was defined as a fall onto an outstretched hand from ground level.

### Radiological measurements

**Fig. 1:**
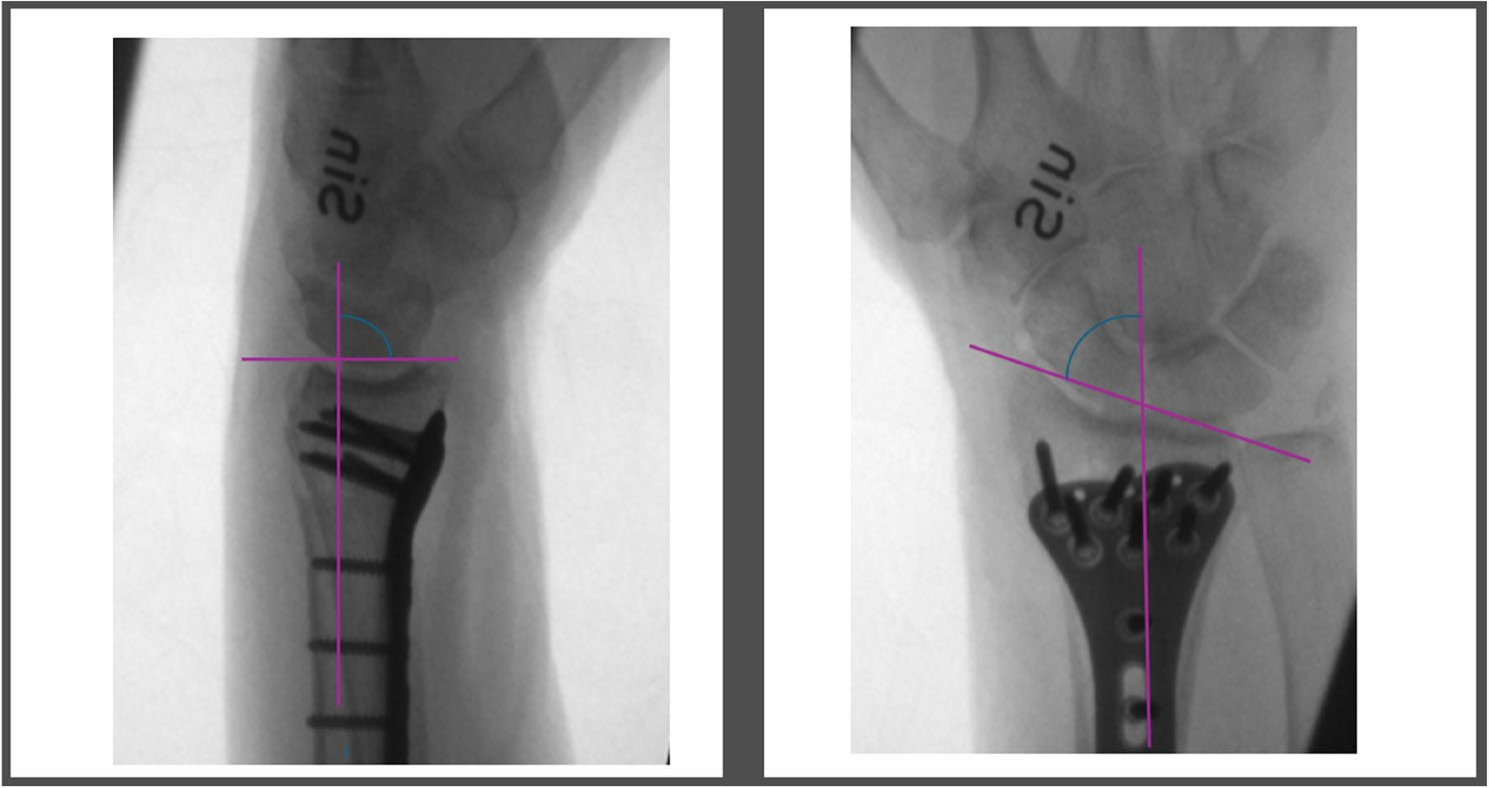
Assessment of dorsal and radial tilt on lateral and anteroposterior radiographs [14]

All patients underwent radiographic imaging pre- and postoperatively. Dorsal tilt was assessed using lateral projections of the distal radius. Measurements were performed by a medical student under supervision of the first author. Enterprise XERO Viewer software (version 8.1.4.191; Aqua Healthcare) was used for all measurements. The angle of dorsal tilt was determined by drawing three reference lines: the first along the longitudinal axis of the radius, the second perpendicular to this axis, and the third connecting the volar and dorsal margins of the articular surface. The intersection of these lines defined the dorsal tilt angle, assessed from a 0° reference point to minimize measurement variability caused by anatomical variations in volar tilt. A visual representation of the measurement technique is provided in Fig. 1.

### Primary outcomes

- Operation time (defined from incision to skin closure).
- Number of surgeons.

### Secondary outcomes

- Radiological outcome (postoperative alignment assessed on final radiograph).
- Complications (e.g., infection, malunion, reoperation).
- Patient characteristics.

### Statistical analysis

The normality of continuous variables was assessed using the Shapiro–Wilk test and visual inspection of histograms and Q–Q plots. When data were not normally distributed, non-parametric analysis was performed using the Mann–Whitney U test. Comparisons between single-surgeon and dual-surgeon groups were made using independent t-tests for continuous variables and chi-squared tests for categorical variables. A p-value of < 0.05 was considered statistically significant. Data analysis was performed using version 30.0.0 of SPSS (IBM Corp., Armonk, NY, USA).

## Results

### Baseline characteristics

**Fig. 2:**
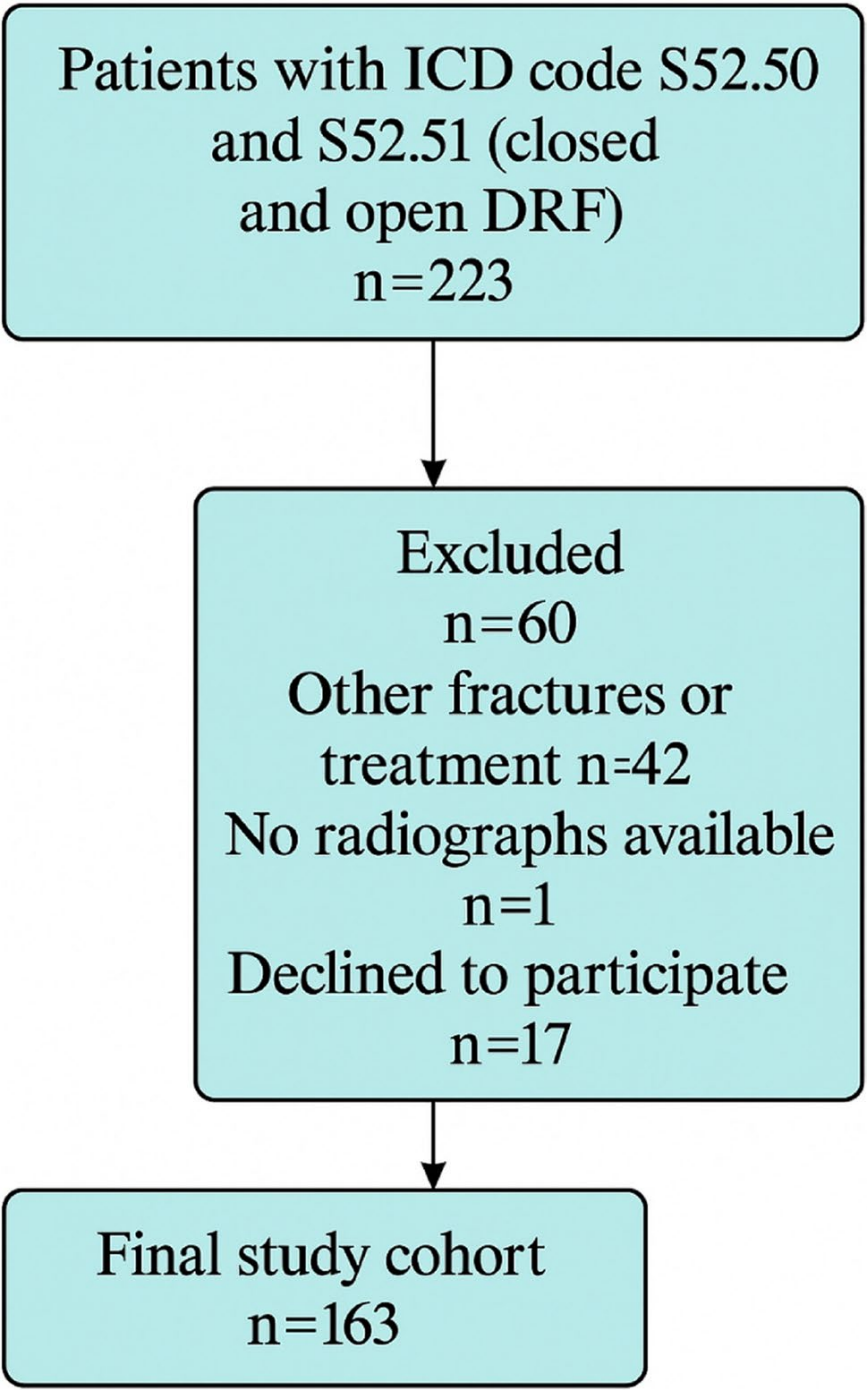
Flowchart of patients included in the study

A total of 223 patients were identified, of whom 60 were excluded for various reasons (Fig. 2), resulting in a final study cohort of 163 patients: 126 women and 37 men. The mean age was 63 years (range: 18–93), and 61 of the cohort were of working age. There were 17 high-energy trauma patients (mean age: 41) and 146 low-energy trauma patients (mean age: 65). The distribution of fracture types is shown in Table 2.

**Table 2:** Fracture type

Fracture type	Total	Single surgeon *n* (%)	Dual surgeons *n* (%)	Chi-square test
A2	53	31 (58.5)	22 (41.5)	Not significant
A3	27	13 (48.1)	14 (51.9)	Not significant
B1	3	3 (100.0)	0 (0.0)	Not significant
C1	10	5 (50.0)	5 (50.0)	Not significant
C2	40	20 (50.0)	20 (50.0)	Not significant
C3	30	18 (60.0)	12 (40.0)	Not significant

### Primary outcome: operation time

Table 3 shows the average operation times for both single-surgeon and dual-surgeon groups, presented with and without the inclusion of level 1 (least experienced) surgeons, and specifically for complex C-type fractures. In the single-surgeon group (*n* = 90), 41% of procedures were performed by level 3 surgeons, 58% by level 2 surgeons, and 1% by level 1 surgeons. In the dual-surgeon group (*n* = 73), the operating surgeon was a level 3 surgeon in 34% of cases, a level 2 surgeon in 47%, and a level 1 surgeon in 19%. The assisting surgeon (*n* = 88) was a level 3 surgeon in 39%, a level 2 surgeon in 30%, and a level 1 surgeon in 32% of cases. The mean operation time was significantly longer when two surgeons participated. However, when class 1 surgeons were excluded, there was no longer any significant difference in mean operation time, either for all fractures or considering only C-type fractures.

**Table 3:** Operation time by single and dual surgeons

	Operation time in min, mean (SD)		*p*-value
	Single surgeon	Dual surgeons	
All fractures	89.3 (31.2)	101.2 (43.1)	0.043*
All fractures Level 1 excluded	88.1 (29.8)	99.5 (54.3)	0.193
C fractures Level 1 excluded	94.1 (32.8)	108.4 (64.8)	0.327

* Statistically significant

### Secondary outcomes

#### Radiological outcomes

Table 4 summarizes the p-values for postoperative malalignment outcomes, comparing dorsal and radial displacement between single-surgeon and dual-surgeon groups. No statistically significant differences were found between groups regarding dorsal or radial malalignment.

**Table 4:** Radiological outcomes

Axis	Single surgeon	Dual surgeons	*p*-value
Dorsal tilt from 0°	3.71 (73; 3.19)	4.18 (90; 3.89)	0.403
Radial inclination	21.79 (73; 3.83)	21.00 (90; 4.25)	0.212

Data are presented in degrees, as mean (n; standard deviation)

#### Complications

Table 5 presents the reoperation rates for both single-surgeon and dual-surgeon groups. No significant difference was observed between the two. There were 17 complications: 1 pseudoarthrosis in the fracture, 2 carpal tunnel syndromes, 2 extensor pollicis longus ruptures, and 12 plate extractions.

**Table 5:** Complication rates by single-surgeon and dual-surgeon group

Group	Reoperation rate	*p*-value
Single surgeon	11%	1.0
Dual surgeons	10%	

#### Sick leave duration

Of the 163 patients in the study cohort, 61 were of working age and needed a sick note. Surgeon experience showed a trend, but not strong significance (*p* = 0.13). Patients operated on by two surgeons had a longer sick leave duration compared to those who underwent single-surgeon operations, but this trend disappeared when class 1 surgeons were excluded. No statistical differences were found related to different fracture classes, only to surgeon experience. Fracture class (AO class) showed no influence on sick leave. Table 6 shows the comparison of sick leave durations between single-surgeon and dual-surgeon groups, both with and without the inclusion of class 1 surgeons.

**Table 6:** Sick leave duration

Group	*p*-value
All cases	0.13
Excluding level 1 surgeons	Not significant

## Discussion

This study analyzed 163 DRFs treated with a volar plate by either one or two surgeons. Operation time was significantly shorter in the single-surgeon group. However, none of the secondary outcomes, including radiological results, showed significant differences between the groups. These findings may be influenced by various factors. At first glance, one might expect that dual-surgeon procedures would reduce operation time, as the surgeons could assist each other and operate four-handed. Conversely, there may be a teaching component in dual-surgeon procedures that could counterbalance this potential time advantage.

While several studies have explored these dynamics in other surgical fields, none have specifically focused on DRFs. For example, studies in spine surgery showed reduced blood loss and reduced hospital stay without increased operation time in dual-surgeon procedures [15]. Another study on spine surgery showed that two less-experienced surgeons did not have inferior outcomes regarding operation time and complications compared to a single more-experienced surgeon [16]. Unfortunately, that study did not report the level of experience in terms of the criteria presented by Tang and Giddins [13], and it is therefore rather difficult to make comparison with the present results. A meta-analysis by Daher et al. supports the hypothesis that dual-surgeon procedures lead to shorted operation time, but the effect on cost effectiveness remains unclear [17].

When considering the AO classification, the significant difference in operation times between the groups diminished in this study, particularly in AO C-type fractures. The increased complexity of these fractures might support the hypothesis that dual-surgeon procedures could help reduce operation times in more complex cases. Nevertheless, the single-surgeon group was still faster, with no negative impact on secondary outcomes such as radiographic results, complications, or sick leave. Other studies on the impact of fracture type indicate that the more complex the fracture, the less favorable the outcome [18]. Dual-surgeon operations may offer teaching opportunities for resident surgeons, but the hypothetical effect on operative time is difficult to measure. In addition, it can be difficult to draw a clear line between “teaching operations” and complex cases where an experienced surgeon needs an assistant but performs the procedure. Some cases may be combined where a junior surgeon starts the operation but the more experienced surgeon takes over during the procedure due to fracture complexity.

No significant difference in complications was observed between the single-surgeon and dual-surgeon groups, which is consistent with a meta-analysis in spine surgery comparing both approaches [19].

### Weaknesses and strengths

Several limitations must be considered when interpreting the present results. The study design was based exclusively on retrospective chart reviews, and did not incorporate patient-reported outcome measures. The inclusion of such measures would have strengthened the findings and potentially identified patients with suboptimal outcomes that did not meet the threshold for surgical revision. Furthermore, as treatment was not randomized, it can´t be excluded that complex fractures were more likely to be operated by a more experienced surgeon or two surgeons.

However, this study covered a full calendar year and included all DRFs (*n* = 163) treated at a regional hospital during that period, which provides a representative overview of clinical practice. Additionally, both the experience level of the surgeons and the fracture classification were accounted for in the analysis.

## Conclusion

The involvement of two surgeons is not necessarily associated with reduced operation time in the treatment of DRF. Furthermore, radiological outcomes and complication rates appear similar regardless of the number of surgeons. These findings may support more efficient allocation of surgical resources in hospital settings.

## Data Availability

The dataset supporting the conclusions of this article is available on request to the corresponding author.

## Abbreviations

DRF: Distal radius fracture
ICD: 10–International Classification of Diseases, 10th Revision
AO/OTA: Arbeitsgemeinschaft für Osteosynthesefragen/Orthopaedic Trauma Association
